# Membrane Nanoscopic Organization of D2L Dopamine Receptor Probed by Quantum Dot Tracking

**DOI:** 10.3390/membranes11080578

**Published:** 2021-07-30

**Authors:** Oleg Kovtun, Ruben Torres, Laurel G. Bellocchio, Sandra Jean Rosenthal

**Affiliations:** 1 Department of Chemistry, Vanderbilt University, Nashville, TN 37235, USA; ruben.torres@vanderbilt.edu (R.T.); laurel.g.bellocchio@vanderbilt.edu (L.G.B.); 2 Vanderbilt Institute of Chemical Biology, Vanderbilt University, Nashville, TN 37235, USA; 3 Department of Pharmacology, Vanderbilt University, Nashville, TN 37235, USA; 4 Department of Chemical and Biomolecular Engineering, Vanderbilt University, Nashville, TN 37235, USA; 5 Vanderbilt Institute of Nanoscale Science and Engineering, Vanderbilt University, Nashville, TN 37235, USA

**Keywords:** quantum dot, G protein-coupled receptor, D2 dopamine receptor, single particle tracking, super-resolution microscopy, photoactivation localization microscopy, schizophrenia, neuropsychiatric disorders, membrane nanodomain, nanoclustering

## Abstract

The role of lateral mobility and nanodomain organization of G protein-coupled receptors in modulating subcellular signaling has been under increasing scrutiny. Investigation of D2 dopamine receptor diffusion dynamics is of particular interest, as these receptors have been linked to altered neurotransmission in affective disorders and represent the primary target for commonly prescribed antipsychotics. Here, we applied our single quantum dot tracking approach to decipher intrinsic diffusion patterns of the wild-type long isoform of the D2 dopamine receptor and its genetic variants previously identified in several cohorts of schizophrenia patients. We identified a subtle decrease in the diffusion rate of the Val96Ala mutant that parallels its previously reported reduced affinity for potent neuroleptics clozapine and chlorpromazine. Slower Val96Ala variant diffusion was not accompanied by a change in receptor-receptor transient interactions as defined by the diffraction-limited quantum dot colocalization events. In addition, we implemented a Voronoї tessellation-based algorithm to compare nanoclustering of the D2 dopamine receptor to the dominant anionic phospholipid phosphatidylinositol 4,5-bisphosphate in the plasma membrane of live cells.

## 1. Introduction

G protein-coupled receptors (GPCRs) are the largest family of transmembrane (TM) protein receptors that mediate a diverse array of subcellular signaling responses and are the targets of a third of all Food and Drug Administration (FDA)-approved drugs [1,2]. GPCR-mediated signaling in response to activation via hormones, ions, neurotransmitters, or photons proceeds through heterotrimeric G protein pathways and G protein-independent routes, including arrestin recruitment [1,2,3,4,5]. The past decade has witnessed unprecedented progress in our understanding of the 3D structure of GPCRs and GPCR-transducer complexes as well as structural transitions that occur over the time course of activated GPCR signaling [3,4,6]. There has also been a growing interest in the lateral organization of GPCRs at the cell surface, as advances in single-molecule fluorescence microscopy and single-particle tracking (SPT) have enabled direct observation of the stochastic and dynamic behavior of individual GPCRs in real-time [7,8,9,10,11,12]. Current state-of-the-art knowledge derived from SPT studies is that (i) a vast majority of GPCRs maintain a diffusible surface pool [13], (ii) activation status controls mobility [9,10,14], (iii) agonist-dependent global decrease in the diffusion rate is not dependent on the GPCR subfamily or G protein coupling selectivity [10], and (iv) GPCRs can assemble into transient homo- and heterodimers, although the preponderance and lifetime of such oligomeric assemblies remain controversial [8,9,15,16]. Nevertheless, these results highlight the need for a continued inquiry into one of the most debated features of GPCR signaling—whether receptors are pre-coupled to signal transducers or engage them via random collisions facilitated by the plasma membrane specialized nanodomains [13,17]. Recently, von Zastrow and colleagues proposed that lateral diffusion of activated Class A GPCR μ-opioid receptors into the presynaptic terminals offers localized signal amplification performance that is inherently superior to that achievable by GPCR-transducer pre-coupling [13]. Therefore, achieving precise control over lateral mobility of GPCRs represents an opportunity for the development of improved therapeutics with greater pharmacological and spatiotemporal specificity.

Our recent research focus has been on characterizing lateral mobility and defining its signal transduction implications for the D2 subtype dopamine receptor (D2DR), a Class A GPCR [14,18]. D2 dopamine receptors are involved in the regulation of dopamine-dependent motor function, motivation, cognition, emotion, and neuroendocrine secretion [19,20]. Dysfunction and polymorphisms of D2 dopamine receptors have been linked to various neuropsychiatric disorders, including addiction, schizophrenia, and Parkinson’s disease (PD) [21,22,23,24,25,26]. Consequently, D2 dopamine receptors are the primary target for both typical and atypical antipsychotic medications as well as anti-parkinsonics used to manage PD [25,27,28,29]. D2 dopamine receptors exist as two alternative splice variants—long isoform (D2L) and short isoform (D2S), which differ in a 29-amino acid insert in the third intracellular loop [30,31]. A popular strategy to enable SPT of dopamine receptors and GPCRs, in general, involves the fusion of an epitope tag (e.g., hemagglutinin (HA; YPYDVPDYA), FLAG (DYKDDDDK), SNAP) to the extracellular N terminus, where it is well tolerated [10,14,32,33,34,35]. We adopted this strategy in our previous report by targeting the HA-D2L receptor construct with a high-affinity biotinylated anti-HA-antibody fragment (Fab) and streptavidin-conjugated quantum dots (SavQdots) [14]. The use of quantum dots, a colloquial term for nanometer-sized semiconductor crystals, allowed us to capitalize on their unique photophysical properties [36,37], including excellent photostability and high brightness, whereas relying on the anti-HA-Fab as the off-site targeting unit enabled monitoring dynamic changes in response to substrate binding. We demonstrated that agonist activation led to a substantial decrease in the diffusion rate of Qdot-tagged D2L receptors. We also observed that a functionally impaired Ser311Cys D2L variant exhibited an attenuated dynamic response to agonist stimulation.

Here, we sought to build upon the previous report and applied our Qdot-based SPT strategy at an increased temporal resolution (30 Hz) to explore whether additional genetic variants of D2 dopamine receptors displayed disrupted diffusion patterns under basal conditions. In addition to wild-type (WT) and Ser311Cys D2L receptors, we examined the Pro310Ser D2L variant, previously shown to confer a risk for schizophrenia in family-based associated studies of Han Chinese in Taiwan, and Val96Ala D2L, a less commonly studied naturally-occurring variant [26]. The substitutions of Pro310 and Ser311 are located in the intracellular cytoplasmic loop 3 (ICL3) and were shown to impair G protein-mediated adenylyl cyclase inhibition by the activated D2 receptors, whereas the substitution of Val96 is located in TM domain 2 proximal to the ligand-binding pocket and was demonstrated to influence the functional response of D2 dopamine receptors to blockade with antipsychotics [20,24,38]. We applied a rigorous diffusion analysis to Qdot-D2L trajectories and found that the Val96Ala D2L transiently expressed in HEK-293T cells displayed subtly perturbed diffusion patterns that differed from the wild-type receptor and mutants bearing the substitution in the ICL3 region. Furthermore, we demonstrated that the slower diffusion rate of the Val96Ala variant was not dependent upon the frequency of Qdot-Qdot merge-and-split events, conventionally defined as protein-protein interaction in the diffraction-limited, single-molecule tracking studies [9,32,39,40]. Finally, we applied a Voronoї tessellation-based algorithm [41,42] to shed light on the degree of D2L receptor nanoclustering in the plasma membrane of live cells compared to the widely expressed anionic phospholipid phosphatidylinositol 4,5-bisphosphate (PIP_2_).

## 2. Materials and Methods

### 2.1. Materials

DMEM, fetal bovine serum, penicillin/streptomycin, DMEM FluoroBrite™ live-cell imaging medium, Lipofectamine 3000, CellMask™ Deep Red Plasma Membrane (PM) stain, and SavQdot (emission max at 655 nm) were purchased from ThermoFisher Scientific (Waltham, MA, USA). Poly-D-lysine hydrobromide (mol wt. 70,000–150,000 Da), bovine serum albumin (BSA), and anti-HA-Biotin (High-Affinity 3F10 clone) from rat IgG1 were purchased from Sigma-Aldrich (Saint Louis, MO, USA). 35-mm uncoated No. 1.5 coverslip-bottomed dishes were purchased from MatTek. pcDNA3.1-D2L-3xHA was acquired from the cDNA Resource Center (DRD020TN00) at Bloomsburg University, Bloomsburg, PA, USA 17815. To generate receptor mutants, the cDNA clone of HA-tagged D2L was used as a template for site-directed mutagenesis using the QuikChange method. The mutant plasmids were sequenced to confirm the desired mutations. pmEos2-N1 PH-PLCd (wild-type) (encoding mEos2-fused PH-PLCδ [43]) was a gift from Frederic Meunier (Addgene plasmid # 162877; http://n2t.net/addgene:162877, accessed on 15 January 2021; RRID:Addgene_162877). MATLAB version 2017b was used for data analysis and figure preparation. ImageJ version 1.53c, TrackMate version 6.02, and u-track version 2.2.0 were used for image processing and trajectory reconstruction. Figures were assembled in Inkscape version 0.92.4.

### 2.2. Cell Culture and Transfections

HEK-293T cells were grown in a complete medium (DMEM with 2 mM glutamine, 10% FBS, 1% pen/strep) in a 37 °C incubator with 5% CO_2_. Cells were seeded in poly-D-lysine coated (1 hr at 37 °C) MatTek dishes at an appropriate density to obtain a subconfluent monolayer and grown for 24 h in the complete growth medium. Then the cells were transiently transfected with 500 ng of the appropriate DNA per MatTek dish using Lipofectamine 3000 according to the manufacturer’s instructions.

### 2.3. Single Qdot Tracking via Spinning Disk Confocal Microscopy

Qdot labeling was implemented via a two-step protocol. After the cells were allowed 24 h to achieve receptor expression, labeling was carried out by first incubating the cells with anti-HA-Fab-biotin at 0.2 µg/mL for 10 min at 37 °C. Following three washes with warm DMEM FluoroBrite™ (Thermo Fisher Scientific, Waltham, MA, USA), cells were then incubated with 0.05 nM SavQdot655 diluted in warm DMEM FluoroBrite™ supplemented with 1% BSA for 5 min at room temperature, washed three times with warm DMEM FluoroBrite™, and used immediately for time-lapse image series acquisition. Time-lapse image series were obtained on an inverted Nikon-Ti Eclipse microscope system equipped with the Yokogawa CSU-X1 spinning disk confocal scanner unit, a heated stage, a 60× oil-immersion Plan Apo 1.4 NA objective, and the Andor DU-897 electron-multiplying charged-coupled device (EMCCD) camera (Oxford Instruments Concord, Concord, MA, USA). Qdots were excited using a 405 nm solid-state diode laser (23 mW), and the Qdot emission was collected through the 641 nm (±75 nm) emission filter. CellMask™ Deep Red PM molecules were excited using the 647 nm laser line (150 mW), and the emission was collected using the 700 nm (±37 nm) emission filter. Single Qdot tracking was performed at a sampling rate of ~30 Hz (Δt = 0.032 s) for 2000 frames. 

### 2.4. Photoactivation Localization Microscopy of mEos2-Fused PH-PLCδ

Time-lapse image series were obtained on an inverted Nikon-Ti Eclipse microscope system equipped with the heated stage, a 60× oil-immersion Plan Apo 1.4 NA objective, and the Andor Zyla 4.2 charged metal oxide semiconductor camera. Individual photoconverted mEos2 molecules were excited using a 561 nm solid-state diode laser (85 mW) in total internal reflection fluorescence (TIRF) mode, and the emission was collected through the 603 nm (±15 nm) emission filter. Photoconversion was achieved by simultaneously illuminating the sample with the 405 nm laser (23 mW) at 5% laser power. The green form of mEos2 molecules was excited using the 488 nm line (65 mW), and the emission was collected using the 525 nm (±25 nm) emission filter. sptPALM was performed at an acquisition rate of 20 Hz for 3000 frames.

### 2.5. Trajectory Reconstruction and Diffusion Analysis

For objective comparison, several popular open-access algorithms were employed to determine the center position of individual Qdots with sub-pixel accuracy (spatial detection stage), connect obtained Qdot coordinates into continuous trajectory segments (temporal detection stage), and estimate the diffusion coefficient for individual trajectories [44,45]. Qdot-D2L trajectories were reconstructed via (1) Crocker–Weeks algorithm that is based on work by Crocker and Grier [46], (2) u-track suite developed by Jaqaman et al. [47] (only trajectories of blinking Qdots with a minimum duration of 50 frames were retained for diffusion analysis), and (3) ImageJ TrackMate plug-in developed by Tinevez et al. [48]. Qdot localization was achieved using the following settings: Gaussian θ = 2 pixels and α = 0.01 for u-track; spatial band-pass filter of (1,4) and peak search with the intensity threshold of 20 and size of 5 pixels for the Crocker–Weeks algorithm; particle size of 1 μm with the threshold of 25 for the TrackMate. Individual Qdot positions were reconstructed into continuous segments using a maximum gap of 10 frames for all algorithms and a maximum displacement of 5 pixels (Crocker-Weeks) or 1 μm (TrackMate). To close gaps and link trajectory segments in u-track, the default settings for the Brownian search radius were used (Brownian search radius upper boundary: 5 pixels; multiplication factor for search radius calculation: 3; number of frames for the nearest neighbor search radius expansion: 11). Only trajectories of blinking Qdots (i.e., containing position gaps due to fluorescence intermittency) with a minimum duration of 50 frames were used for subsequent diffusion analysis. Diffusion coefficients for individual filtered trajectories were determined via Maximum Likelihood Estimation (MLE) algorithm [49,50] with the motion blur coefficient of 0 (minimum allowed value) or 0.25 (maximum allowed value) and mean square displacement (MSD)-based method [51,52]. For each trajectory, MSD was computed as follows:

(1) $$\mathrm{MSD}\left(t\right)=\frac{1}{N-n}\overset{N-n-1}{\underset{j=0}{\sum }}\left\{{\left[x\left(j\delta t+n\delta t\right)-x\left(j\delta t\right)\right]}^{2}+{\left[y\left(j\delta t+n\delta t\right)-y\left(j\delta t\right)\right]}^{2}\right\}$$

where δt is the temporal resolution of the acquisition device, (x(jδt), y(jδt)) is the particle coordinate at t = jδt, and N is the number of total frames recorded for an individual particle. Prior to MSD computation, individual trajectories were either reindexed with a continuous time vector to close the gaps caused by blinking or analyzed with gaps present. The diffusion coefficient D_2–5_ was calculated from the slope of the first 2–5 points of the MSD plot versus time with the equation:

(2) $$\mathrm{MSD}\left(t\right)=4D_{2-5}t+4\sigma_{x}^{2}$$

where σ_x_ is the spot localization accuracy in one direction (i.e., $4\sigma_{x}^{2}$ is the ordinate at the origin of the linear fit). Trajectories of Qdots greater than the previously determined immobile particle threshold 5 × 10^−4^ μm^2^/s [14,53] were used for statistical comparison via the nonparametric Mann–Whitney U test and Kolmogorov–Smirnov test. Merge and split events occurring in Qdot time-lapse image series were determined by using the fully functional linear assignment problem tracker in TrackMate [47,48] while allowing the detection of Qdot signal splitting and merging with a maximum displacement of 1 μm.

### 2.6. Cluster Identification Using Voronoї Tessellation 

Super-resolved maps of mEos2 and Qdot localizations were reconstructed using the ThunderSTORM plug-in [54] in ImageJ. For mEos2, single planes were processed using a lowered Gaussian filter with σ = 1.6 pixels, and individual emitters were localized via non-maximum suppression with a peak intensity threshold of 6 and a dilation radius of 3 pixels. For Qdots, single planes were processed using a B-Spline wavelet filter with B-Spline order of 3 and B-Spline scale of 2.0, with individual Qdots localized via non-maximum suppression with a peak intensity threshold of 20 and a dilation radius of 3 pixels. Voronoї diagrams for data sets of (x,y) localizations corresponding to the individual time-lapse image series were constructed by first computing a two-dimensional Delaunay triangulation using the built-in MATLAB function delaunayTriangulation and then determining Voronoї vertices and Voronoї regions of the points in a Delaunay triangulation array using the built-in MATLAB function voronoiDiagram. Cluster search was achieved by first omitting Voronoї cells with an area above the 50th percentile threshold computed for individual time-lapse image series and then identifying regions of at least 10 connected remaining Voronoї cells. The individual cluster radius was calculated as $radius=\surd \frac{Area}{\pi }$. 

## 3. Results and Discussion

### 3.1. Qdot Tracking of D2L Receptors

**Figure 1:**
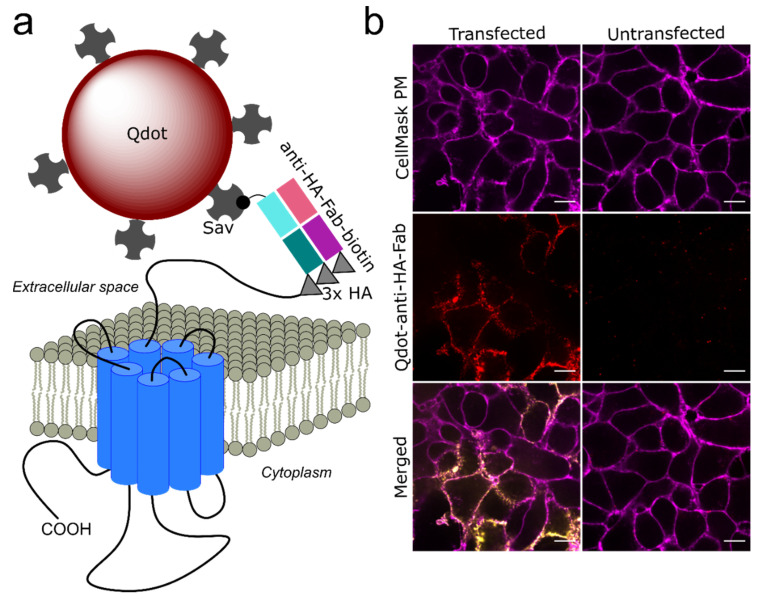
Qdot labeling of HA-tagged D2L receptors. (**a**) Schematic of the labeling approach is shown here (drawn not to scale). Triple HA-tagged D2L receptors were labeled at the surface of transiently transfected HEK-293T cells with biotinylated anti-HA-Fab and streptavidin-conjugated Qdots; (**b**) Labeling specificity was confirmed by incubating transfected and untransfected HEK-293T cells with anti-HA-Fab-biotin/SavQdot655 in the presence of CellMask PM stain to show the outline and the relative focal plan of imaged cells. Scale bar: 10 μm.

Lateral mobility of D2L dopamine receptors was examined using an engineered D2L construct with a triplet HA epitope tag fused to the extracellular N terminus. Figure 1a shows a schematic of the D2L construct labeled with biotinylated anti-HA-Fab and SavQdots in successive incubations. The N-terminal HA tag was shown to be well-tolerated by D2 dopamine receptors and enabled the detection of surface molecules [14,33]. A similar labeling strategy utilizing an engineered extracellular HA epitope tag was successfully employed for Qdot tracking of cystic fibrosis transmembrane conductance regulator (CFTR) protein [55], serotonin 1A receptor [34], and epidermal growth factor receptor [39,56]. Selective Qdot labeling of epitope-tagged D2L transiently expressed in HEK-293T cells was confirmed, with only a few Qdot puncta detected in the field of view in untransfected cells incubated with anti-HA-Fab and SavQdots (Figure 1b). Qdot-D2L were imaged in the presence of the CellMask PM stain to determine whether a significant number of Qdot-labeled D2L receptors localized to intracellular compartments and whether surface dynamic events were primarily observed. Figure 1b shows limited internalization of Qdot-D2L complexes in live HEK-293T cells post-labeling. Such endocytic behavior of D2L is in agreement with previous studies that demonstrated different sensitivity of D2S versus D2L to surface loss under stimulated and unstimulated conditions [57]. The consensus is that D2L receptors exhibit a significantly lower extent of internalization that also proceeds more slowly compared to the D2S isoform. The presence of the long intracellular loop 3 in the D2L sequence has been hypothesized to attenuate D2L desensitization via endocytic trafficking [57], thus enabling direct monitoring of D2L surface diffusion dynamics with greater confidence in contrast to D2S receptors.

### 3.2. Comparison of Algorithms for Trajectory Reconstruction and Diffusion Coefficient Determination

**Figure 2:**
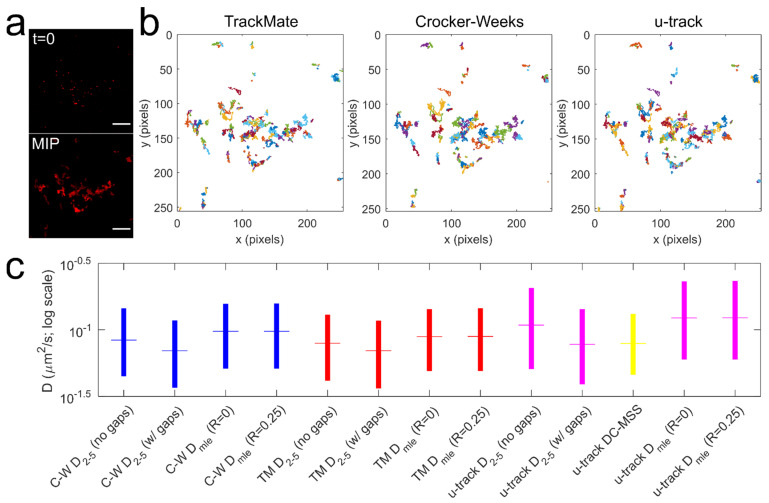
Tracking and diffusion analysis of Qdot-tagged wild-type D2L receptors. (**a**) An example single-plane view of Qdot-D2L localized at the surface of HEK-293T cells (top; scale bar: 10 μm); a maximum intensity projection (MIP) of 2000 frames acquired at 30 Hz and generated in Image J (bottom); (**b**) trajectories reconstructed from a single time-lapse image series via three different open-access, popular algorithms are shown here (1 pixel = 0.225 μm); (**c**) diffusion coefficient distributions of Qdot-tagged, wild-type D2L receptors (n = 10 movies) for each computation method are shown as box plots with the colored horizontal line in the box corresponding to the median value and the vertical edges of the colored box corresponding to the 25–75% interquartile range (IQR); the following median D values were obtained for mobile trajectories (D > 5 × 10^−4^ μm^2^/s): C–W D_2–5_ (no gaps) = 0.084 μm^2^/s (0.045–0.145 IQR, N = 3133 tracks), C-W D_2–5_ (with gaps) = 0.070 μm^2^/s (0.037–0.118 IQR, N = 3105 tracks), C–W D_MLE_ (R = 0) = 0.097 μm^2^/s (0.051–0.157 IQR, N = 3131 tracks), C–W D_MLE_ (R = 0.25) = 0.097 μm^2^/s (0.051–0.157 IQR, N = 3131 tracks), TM D_2–5_ (no gaps) = 0.079 μm^2^/s (0.042–0.130 IQR, N = 3978 tracks), TM D_2–5_ (with gaps) = 0.070 μm^2^/s (0.036–0.117 IQR, N = 3943 tracks), TM D_MLE_ (R = 0) = 0.089 μm^2^/s (0.049–0.143 IQR, N = 3984 tracks), TM D_MLE_ (R = 0.25) = 0.089 μm^2^/s (0.049–0.145 IQR, N = 3984 tracks), u-track D_2–5_ (no gaps) = 0.108 μm^2^/s (0.051–0.207 IQR, N = 3832 tracks), u-track D_2–5_ (with gaps) = 0.078 μm^2^/s (0.039–0.143 IQR, N = 3752 tracks), u-track DC-MSS D = 0.079 μm^2^/s (0.046–0.132 IQR, N = 3965 tracks), u-track D_MLE_ (R = 0) = 0.123 μm^2^/s (0.060–0.231 IQR, N = 3879 tracks), u-track D_MLE_ (R = 0.25) = 0.123 μm^2^/s (0.060–0.233 IQR, N = 3752 tracks).

Trajectories of individual Qdot-D2L puncta were recorded in the coverslip-plasma membrane focal plane with a sampling rate of ~30 Hz (Δt = 0.032 s) (Figure 2a). Presently, numerous particle detection and tracking software suites exist, yet there is no standardized set of field-wide guidelines for point emitter detection and trajectory reconstruction [58]. Moreover, accurate diffusion rate determination and motion type identification remain controversial topics [49]. Furthermore, Qdot detection and trajectory reconstruction may be complicated by photoluminescence (PL) intermittency, PL sensitivity to pH or ionic strength of the imaging buffer, and the sampling rate of time-lapse imaging [37,59], even though Qdots are a robust photon emitter and are uniquely suited to facilitate SPT with a high signal-to-background ratio in two- and three-dimensional biological specimens [37,60]. In particular, PL intermittency or blinking of single Qdots poses challenges for establishing accurate frame-to-frame positional correspondence [47]. Commercially available Qdots may also be susceptible to PL degradation in an oxygen-rich environment [60]. Here, we sought to compare three popular open-source detection and tracking algorithms (u-track developed in the Danuser lab [47], ImageJ plug-in TrackMate, and MATLAB-based Crocker–Weeks (C–W) routines originally developed by Crocker and Grier [46]) in combination with three methods of diffusion coefficient determination for individual Qdot trajectories. The u-track suite relies on fitting local signal maxima with a Gaussian kernel (Gaussian Mixture Model) at the spatial (detection) stage; in a standard TrackMate protocol one first applies a Laplacian of Gaussian filter to each frame of the image series followed by a quadratic fitting of local maxima to achieve sub-pixel accuracy; C–W algorithm calculates a brightness-weighted centroid of the pixels in a region around local maxima identified in each frame first filtered with a spatial band-pass filter. At the temporal (frame-to-frame linking) stage, u-track and TrackMate both use a globally optimized linear assignment problem (LAP) to provide an accurate solution to multiple track segment linkage, whereas the C–W script determines the most likely assignment of particle labels from one frame to the next by maximizing $P\left(\left\{\delta_{i}\right\}|\tau \right)$ as follows:

(3) $$P\left(\left\{\delta_{i}\right\}|\tau \right)={\left(\frac{1}{4\pi \mathrm{Dt}}\right)}^{N}\exp\left(-\overset{N}{\underset{i=1}{\sum }}\frac{\delta_{i}^{2}}{4D\tau }\right)$$

where P is the probability that a single Brownian particle i in an ensemble of N noninteracting identical particles will diffuse a distance δ in the plane in time τ with a self-diffusion coefficient D. Figure 2b shows trajectories of Qdot-D2L reconstructed for a representative time-lapse image series via the aforementioned algorithms. At the trajectory analysis stage, the u-track suite employs a built-in divide-and-conquer moment scaling spectrum (DC-MSS) algorithm that computes normal diffusion coefficient by fitting the first five (or fewer) points of the log-log plot of the second moment of the displacement distribution (also known as MSD) versus time and dividing the obtained slope by 4 [61]. Another popular method of determining the diffusion coefficient is by fitting the first 2–5 points of MSD versus time curve, the most linear region for different types of diffusive behavior and less likely to be influenced by the localization error compared to the first point [52]. On the other hand, Berglund’s MLE algorithm [50] is based on numerically maximizing the log-likelihood function (in frequency domain) of the matrix of the observed particle’s single-step displacements that are a function of particle’s diffusion coefficient D and static localization noise σ. The graphical summary of the results of diffusion analysis of Qdot-tagged wild-type D2L receptors via different method combinations is shown in Figure 2c, and the key findings of this comparison are listed below:

1. The D_2–5_ diffusion coefficient of Qdot-D2L trajectories that were rendered continuous by removing the gaps due to missing Qdot position (Qdot blinking or out-of-focus drift) was significantly faster (*** *p* < 0.0001, Mann-Whitney U test) than the D_2–5_ diffusion coefficient of Qdot-D2L trajectories that contained gaps for all trajectory reconstruction algorithms;
2. The motion blur coefficient R (dynamic error in the particle’s position due to frame averaging) in the MLE algorithm had no significant effect on the *D_MLE_* diffusion coefficient for all trajectory reconstruction algorithms (R_min_ = 0 versus R_max_ = 0.25; *p* > 0.05, Mann–Whitney U test);
3. The MLE algorithm consistently yielded a greater diffusion rate than the methods that rely on fitting the first few points of the MSD-time curve or its log-log form (*** *p* < 0.0001, Mann–Whitney U test).
4. The diffusion rate of Qdot-D2L determined via widely used u-track package was only not statistically different from D_2–5_ determined for u-track trajectories that contained gaps due to missing Qdot positions and CW trajectories that were rendered continuous (*p* > 0.05, Mann-Whitney U test).

To sum up, the determination of the diffusion coefficient for single-particle trajectories was found to be sensitive to the method employed, thus supporting the need for implementation of standardized field-wide guidelines that will ultimately permit direct comparison of published diffusion rate distributions for various tracked targets. 

### 3.3. Diffusion Dynamics of D2L Variants

**Figure 3:**
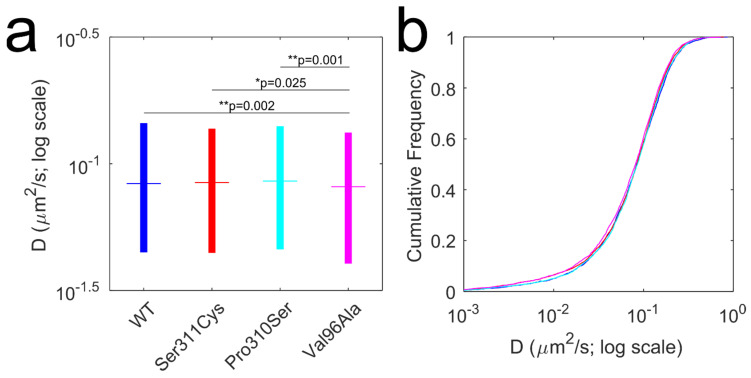
Comparison of the Qdot-tagged D2L receptor variant lateral mobility. (**a**) C–W D_2-5_ (no gaps) diffusion coefficients were determined for four D2L variants tracked and the box plots with 25–75% IQR shown here. The following median D values were determined for mobile D2L variants: D_WT_ = 0.084 μm^2^/s (0.045–0.145 IQR, N = 3133 tracks), D_Ser311Cys_ = 0.084 μm^2^/s (0.045–0.137 IQR, N = 4772 tracks from 10 movies), D_Pro310Ser_ = 0.085 μm^2^/s (0.046–0.141 IQR, N = 3126 tracks from 10 movies), D_Val96Ala_ = 0.097 μm^2^/s (0.040–0.133 IQR, N = 3753 tracks from 10 movies). Statistical significance of pairwise comparison using the nonparametric Mann–Whitney U test is displayed as p values within the figure panel. (**b**) The cumulative frequency plot shows another representation of diffusion coefficient distributions for four D2L variants tracked. Pairwise statistical significance via the nonparametric Kolmogorov–Smirnov test revealed that the diffusion rate of Val96Ala variant was significantly different from WT (** *p* = 0.002), Pro310Ser (** *p* = 0.009), and Ser311Cys (* *p* = 0.04). Additionally, the diffusion rate of the Val96Ala D2L variant was found to be significantly different from the wild-type D2L protein for C–W D_2–5_ (with gaps), C–W D_mle_, T-M D_mle_, T-M D_2–5_ (no gaps), and T-M D_2–5_ (with gaps) distributions via both the Mann–Whitney U test and the Kolmogorov–Smirnov test.

**Figure 4:**
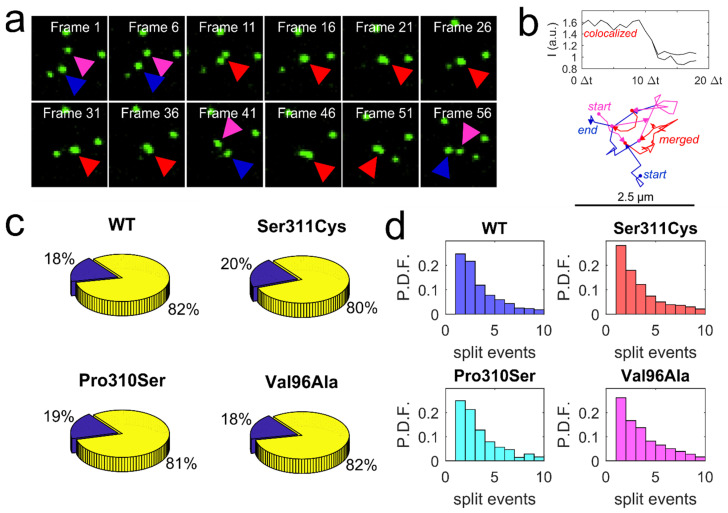
Detection of merging and splitting events in Qdot-D2L trajectories. (**a**) A representative time-lapse series shows two separated Qdots (blue and magenta arrows) transiently merging into a single spot (red arrow) with higher total intensity. (**b**) An example intensity profile of an observed merged (colocalized) Qdot spot splitting into two separate trajectory segments (top; Δt = 0.1 s); a representative reconstructed trajectory containing a merged (red) track segment is shown here. (**c**) Three-dimensional pie charts show a fraction of trajectories undergoing merging and splitting (blue wedge) for each D2L coding variant tracked (438 out of 2483 tracks with more than 50 detected spots in length for WT; 611 out of 3468 tracks (>50 spots in length) for Val96Ala; 493 out of 2659 tracks (>50 spots in length) for Pro310Ser; 797 out of 3944 tracks (>50 spots in length) for Ser311Cys; n = 10 movies acquired at 30 Hz for 2000 frames and previously used for diffusion rate analysis). (**d**) Histograms of the number of splitting events per M&S-positive trajectories identified in (**c**) are shown here.

According to the dopaminergic hypothesis of affective disorders [62,63], symptom expression is dictated by dysregulation of the dopaminergic neurotransmission via key molecular actors—dopamine transporter and dopamine receptors. Over the years, particular emphasis has been placed on studying the association between the genetic variation in the D2 dopamine receptor locus and schizophrenia risk, as the clinical response of antipsychotic drugs is linked to receptor occupancy [26,27,64]. A recent genome-wide association study indicated that common variants of D2 dopamine receptors conferred an increased risk of schizophrenia [65]; preliminary evidence suggests that genetic variation in D2 dopamine receptors may alter antipsychotic drug potency and disrupt dynamic interactions with the receptor binding partners [26,38]. In our previous report, we demonstrated that a naturally occurring, functionally deficient D2L Ser311Cys variant displayed an attenuated diffusional slowing in response to agonist stimulation in comparison to the wild-type receptor [14]. Here, we sought to extend our Qdot tracking approach to an additional two, less frequent and less widely studied, D2 dopamine receptor variants—Pro310Ser and Val96Ala. Our principal goal was to explore whether the relative location of the amino acid substitution—TM2 in the proximity of the ligand-binding site (Val96Ala) versus ICL3 (Pro310Ser and Ser311Cys)—had a pronounced effect on the membrane diffusion dynamics of D2L under basal conditions. Diffusion analysis of Qdot-labeled D2L variants at the surface of transiently transfected HEK-293T cells revealed a subtle, quantitatively small but statistically significant difference (Figure 3)—Val96Ala variant displayed a slower rate of lateral diffusion when compared to the other D2L variants. This finding is consistent with our previous observation that Ser311Cys D2L mutant exhibited normal diffusion patterns under basal conditions. As expected, the Pro310Ser variant examined in this study also showed unperturbed basal diffusion dynamics, suggesting that ICL3 positions 310–311 do not exert significant control over D2L intrinsic motion in the membrane. In contrast, Val96Ala substitution had a greater effect on the Qdot-D2L basal diffusion dynamics, which parallels a previously reported ~50% reduction in binding affinities of dopamine, clozapine, and chlorpromazine to Val96Ala D2 dopamine receptor mutant in vitro [24,38]. It appears that coding variation in the TM2 not only potently regulates ligand binding, but is also involved in defining intrinsic diffusion patterns of the receptor. When inspecting and analyzing individual time-lapse image series, we noticed that a significant fraction of Qdots bound to D2L underwent characteristic merge and split (M&S) events, wherein two diffraction-limited spots of lower intensity merged to form a single, transient spot of higher intensity that subsequently split (Figure 4a). SPT studies typically interpret such particle colocalization/codiffusion as physically interacting molecules, although the diffraction-limited nature of imaging exceeds the molecular scale substantially [9,32]. Another challenge in the interpretation of M&S events is distinguishing between true physical interactions and coincident localizations. Consequently, SPT-based analysis of GPCR oligomeric status remains controversial and requires careful vetting using complementary techniques, such as co-immunoprecipitation, fluorescence resonance energy transfer (FRET), bioluminescence resonance energy transfer (BRET), or proximity ligation assay (PLA) [15,16]. Still, there has been a growing interest in the existence, structure, and functional properties of dopamine receptor homo- and heterooligomeric complexes [21,32,35,66]. Targeting oligomer assemblies of dopamine receptors may potentially yield novel therapeutic avenues with greater specificity and reduced side effects. SPT studies of D2 dopamine receptor oligomerization based on single-channel intensity and/or colocalization have yielded conflicting data on oligomer prevalence and the lifetime of the interaction [32,35]. Extracting oligomeric status of the receptor from single-channel Qdot movies is complicated by larger Qdot size compared to smaller organic dyes, increased trajectory fragmentation due to Qdot photoblinking, and greater variation in relative brightness along with the existence of a significant fraction of non-emissive (“dark”) Qdots within each nanocrystal batch [36,37,67]. Such variability in brightness is particularly problematic, as it does not permit the retrieval of receptor oligomeric state based on the point emitter intensity in contrast to organic dyes. However, the optical detection strategy in our study employed a spinning disk confocal system, which enables dynamic imaging with reduced laser power and thereby reduces fluorophore photobleaching rates as well as, in principle, Qdot photoblinking rates compared to laser scanning and widefield illumination. Therefore, we applied a standard M&S detection algorithm based on the LAP tracker to Qdot-D2L time-lapse image series [47]. Figure 4b shows an example intensity trace of a trajectory containing a resolved split event. Although individual M&S events were readily detected, a relatively high degree of trajectory segment fragmentation [68] due to Qdot photoblinking did not allow us to retrieve the duration (lifetime) of merged (colocalized) trajectory segments with a high degree of confidence. Nevertheless, we were able to quantify the fraction of trajectories containing M&S events for each D2L variant (Figure 4c) and the average number of split events per individual trajectory (Figure 4d). All D2L variants showed a similar preponderance of M&S-positive trajectories ranging from 18% to 20%, with ~6–7 splits on average per M&S-positive trajectory. Although a direct comparison of our data (18% fraction of M&S-positive trajectories for wild-type D2L) to previously published data on D2L oligomerization is not appropriate, Tabor et al. reported a comparable degree of intensity-based dimerization for dye-labeled D2L constructs expressed in CHO cells (30% for Alexa546-labeled SNAP-D2L; 26% for Cy3B-antagonist-labeled SNAP-D2L; 29% for Cy3B-antagonist-labeled unmodified D2L) [35]. Based on our analysis, it can be concluded that the reduced diffusion rate of the Val96Ala D2L variant did not translate to the increased frequency of M&S and the average number of transitions per M&S-positive trajectories. Our future work will seek to employ multicolor Qdot tracking [39] to decipher the lifetime of D2L-D2L transient colocalization-codiffusion events and shed light on the physiological significance of this phenomenon. 

### 3.4. Application of Voronoї Tessellation to Qdot-D2L Trajectories

**Figure 5:**
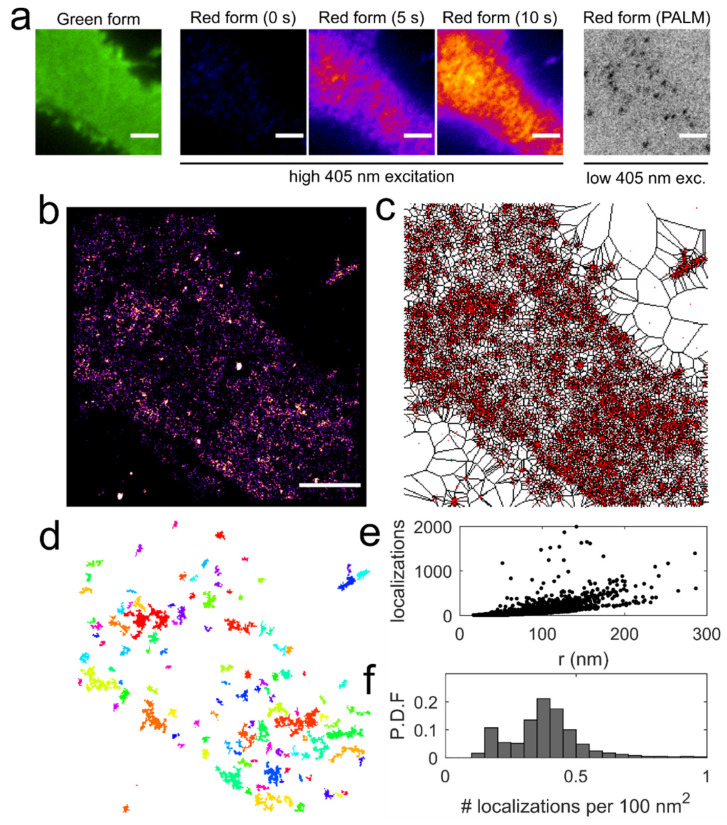
Application of the Voronoї tessellation to detect PIP_2_ membrane nanoclusters. (**a**) A low-resolution TIRF image shows the distribution of the green form of mEos2-PH-PLCδ in an example membrane region (scale bar: 3 μm). Photoconversion of mEos2-PH-PLCδ was confirmed at higher 405 nm laser power (>50%). Super-resolution imaging (sptPALM) of the photoconverted mEos2-PH-PLCδ at sparse surface density was performed at 20 Hz for 3000 frames. (**b**) A super-resolved image of mEos2-PH-PLCδ detections is displayed as an averaged histogram image in ‘Fire’ LUT (scale bar: 3 μm). (**c**) a Voronoї diagram computed for mEos2-PH-PLCδ detections (red) is displayed. (**d**) Individual clusters comprised of connected Voronoї cells were identified. (**e**,**f**) Cluster size (radius), number of localizations per cluster, and localization density per 100 nm^2^ for each cluster were computed for 4246 PIP_2_ clusters (n = 4 movies).

Our final goal of the study was to determine whether Qdot-tagged D2L displayed a clustered distribution at the surface of HEK-293T cells. Although Class A GPCRs are not believed to form discrete nanodomains in the plasma membrane [13], recent super-resolution microscopy evidence indicates that D2 dopamine receptors exist in stable, discrete nanoclusters in the presynaptic terminals of cultured mouse dopaminergic neurons [69]. As a proof-of-concept and a benchmark experiment, we first expressed a photoconvertible fluorescent protein mEos2 fused to the N terminus of the pleckstrin homology domain of phospholipase C delta (mEos2-PH-PLCδ) in HEK-293T cells [43]. PH-PLCδ enabled specific targeting of plasma membrane PIP_2_ at a 1:1 stoichiometry with a K_d_ of ~2 μM and imaging of PIP_2_ nanodomain organization via classical spt-photoactivation localization microscopy (sptPALM) in TIRF mode [70]. PIP_2_ is a dominant inner-leaflet anionic phospholipid involved in a variety of important cellular functions including membrane targeting, cytoskeletal attachment, and neuronal exocytosis [43,71]. Individual molecules of mEos2-PH-PLCδ were detected by stochastically photo-converting mEos2 [72] from the green to red from (Figure 5a) using low 405-nm laser power. Although the exact mechanism of green-to-red photoconversion is still under debate, it was demonstrated that the light-induced cleavage of His62 N^α^-C^α^ bond and the subsequent formation of C^α^-C^β^ bond in the His62 side-chain extends the π-conjugation of the Hi62-Tyr63-Gly64 chromophore triad [73]. Time series (3000 frames) of mEos2-PH-PLCδ detections were acquired at 20 Hz sampling rate, which allowed super-resolution reconstruction with adequate signal-to-noise ratio using the ImageJ ThunderSTORM plug-in (Figure 5b) [54]. To analyze the super-resolved image of PIP_2_ distribution, we applied a Voronoї tessellation algorithm that is based on the construction of a Voronoї diagram for a set of sub-pixel localizations of the point emitter (Figure 5c). The Voronoї diagram subdivides the super-resolved image into polygons centered on individual localizations, such that any point with a given polygon is closer to the localization than to neighbor localizations. This provides a convenient way to characterize the molecular density of a target biomolecule at multiple length scales. The threshold for individual cluster detection was defined as at least 10 connected Voronoї cells (polygons) with an area in the lower 50th percentile (Figure 5d). Radius, number of localizations, and localization density per 100 nm^2^ were computed for each cluster (Figure 5e,f) for further comparison with Qdot-based tessellation data.

**Figure 6:**
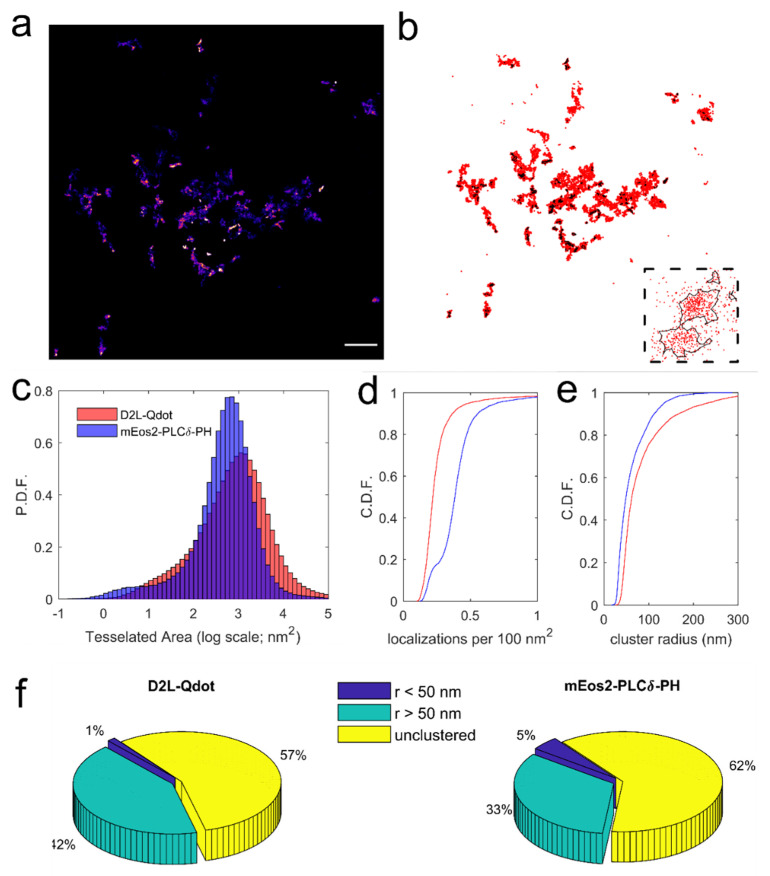
Comparison of Voronoї tessellation-based cluster detection for movies of Qdot-D2L acquired via nonstochastic SPT versus stochastic sptPALM of mEos2-PH-PLCδ. (**a**) A representative ThunderSTORM super-resolved map of Qdot-tagged wild-type D2L localizations is shown (scale bar: 10 μm). (**b**) A Voronoї diagram computed for Qdot-D2L detections (red) is displayed, with identified clusters shown with black contours. (**c**) Histograms of individual Voronoї cell areas are plotted for both Qdot-D2L localizations (1,221,987 detections from 10 movies) and mEos2-PH-PLCδ detections (817,041 detections from 4 movies). (**d**) Cumulative frequency plots of localization density per 100 nm^2^ of identified clusters are shown for Qdot-D2L (red; 4117 clusters) and mEos2-PH-PLCδ (blue; 4246 clusters). (**e**) Cumulative frequency plots of cluster radius are shown for identified localization clusters of Qdot-D2L (red) and mEos2-PH-PLCδ (blue). (**f**) Fractions of Qdot-D2L and mEos2-PH-PLCδ detections found in smaller clusters (r < 50 nm), larger clusters (r > 50 nm), and unclustered form are displayed as three-dimensional pie charts.

Super-resolved images of Qdot-D2L (wild-type receptor) localizations were reconstructed (Figure 6a) using the ThunderSTORM plug-in in a similar manner to mEos2-PH-PLCδ, and individual clusters were detected as described above (Figure 6b). A valid concern arises whether such an analytical combination (ThunderSTORM + Voronoї tessellation), which was designed with stochastic localization data in mind, is appropriate for the analysis of Qdot-bound membrane protein distribution. Indeed, one of the fundamental features of sptPALM and related techniques is the stochastic nature of target localization sampling, whereas Qdot labeling is currently limited to a small subset of membrane proteins that may or may not constitute a representative sample of the entire population at the membrane. Moreover, there has been limited success in developing photo-switchable Qdots for use in stochastic super-resolution microscopy [74]. However, Voronoї tessellation can be applied to a set of sub-pixel localizations obtained for a robust, non-photo-switchable emitter (e.g., Qdot) in a tracking and localization microscopy (TALM) concept [75] that complements sptPALM, wherein longer trajectories yield hidden information about subcellular morphology. One should note that a potential caveat is false-positive cluster detection due to emitter overcounting that arises from repetitive on-off blinking (especially true for immobile targets) [76]. The first step in our analysis was estimating individual Voronoї cell areas for mEos2-PH-PLCδ and Qdot-D2L localization maps, as it allows assessment of changes in emitter localization density in a parameter-free manner when compared to subjective cluster detection [41]. Figure 6c shows histograms of Voronoї cell area distributions for mEos2-PH-PLCδ and Qdot-D2L, with a median area of 552 nm^2^/cell and 854 nm^2^/cell, respectively (*** *p* < 0.0001, Kolmogorov–Smirnov test). Cluster localization density differed significantly for two data sets as well, with 0.43 ± 0.52 mEos2 detections per 100 nm^2^ and 0.27 ± 0.30 Qdot detections per 100 nm^2^ (Figure 6d). Similarly, the average radius of identified clusters was determined to be 61 ± 36 nm for mEos2-PH-PLCδ and 86 ± 62 nm for Qdot-D2L localization data sets. A subsequent review of prior super-resolution studies of PIP_2_ and D2 dopamine receptor nanoclustering and nanodomain distribution revealed conflicting results. Van den Bogaart et al. used a citrine-tagged PH-PLCδ probe in combination with stimulated emission by depletion (STED) microscopy to identify PIP_2_ nanoclusters in the membrane sheets of neuronal PC12 cells with a radius of 36 ± 21 nm [77]. Wang and Richards reported the radius of PIP_2_ nanoclusters to be 32 ± 10 nm in fixed PC12 cells when resolved using Alexa647-anti-PIP_2_ antibody in a STORM experiment [78]. However, Ji et al. demonstrated that sptPALM of iRFP-PAmCherry1-PH-PLCδ revealed a predominantly uniform, homogenous PIP_2_ distribution in the plasma membrane sheets of insulin-releasing INS-1 cells with a small fraction of PIP_2_ localized to domains with an average radius of 192 ± 7 nm [79]. Live-cell imaging of PIP_2_ diffusion dynamics via FRAP of GFP-fused PH-PLCδ in HEK-293 cells, sptPALM of PAmCherry-tagged PH-PLCδ in HEK-293 cells, and sptPALM of mEos2-fused PH-PLCδ in PC12 cells showed that PIP_2_ diffused freely in the membrane (mobile pool of ~80–85%) with limited nanoclustering [4,43,71]. In agreement with these live-cell findings, classification of mEos2-PH-PLCδ detections in our study revealed that the majority of PIP_2_ existed in an unclustered form (62%) or localized to larger nanoclusters with an average radius ≥ 50 nm (33%) in live HEK-293T cells (Figure 6f), with only a small fraction (5%) found in smaller clusters (≤50 nm radius). Super-resolved membrane organization of D2 dopamine receptors at the nanoscale has not been as well-studied as that of PIP_2_ in part due to known problems with specificity and selectivity of dopamine receptor antibodies. Miklosi et al. found evidence of D2 dopamine receptor nanoclustering at the presynaptic and postsynaptic sites of rat hippocampal synaptosomes and cultured hippocampal neurons, although the clusters were less dense and more synaptically dispersed compared to D1 dopamine receptors [80]. Recently, Lycas et al. reported the presence of D2 dopamine autoreceptor nanoclusters in the membrane of cultured mouse dopamine neurons in an antibody-based STORM experiment, showing that a substantial fraction of D2 dopamine receptor detections were localized to clusters of various sizes (radius less or greater than 37.5 nm), shape, and density [69]. However, live-cell SPT experiments showed that both splice variants of the D2 dopamine receptor are predominantly freely diffusing at the surface of transfected cells [14,32,35]. In comparison, cluster analysis of our Qdot-D2L data indicated that the majority (57%) of detections were localized to unclustered regions and the rest of the detections (42%) predominantly populated larger identified clusters with an average radius of 50 nm or more (Figure 6f). Only a small fraction of Qdot-D2L detections were determined to localize to smaller nanoclusters, in agreement with a paucity of evidence supporting Class A GPCR nanoclustering in the plasma membrane [13]. Together, our data suggest that Voronoї-based tessellation analysis can be applied to Qdot localization data sets in order to reveal plasma membrane compartmentalization, although the existence of dopamine receptor nanodomains and nanoclusters under physiologically relevant conditions and their significance in the context of signaling remains controversial. 

## 4. Conclusions

Technological advances in single-molecule optical microscopy over the past two decades have provided a glimpse into the extraordinary complexity of dynamic plasma membrane organization at length and time scales that were previously inaccessible. In particular, perturbed surface diffusion patterns and nanodomain organization of neuronal transmembrane proteins in neuropsychiatric disorders are emerging as an important focus of research inquiry [81]. Here, we extended our previous report and applied a single Qdot tracking paradigm to rigorously characterize the dynamic behavior of the D2L receptor and its naturally occurring genetic variants previously identified in cohorts of patients diagnosed with schizophrenia. We demonstrated that trajectory and diffusion analysis algorithm had a significant effect on the output (diffusion coefficient distributions), thus highlighting the need for a standardized set of field-wide guidelines when quantifying and qualifying diffusion behavior of biomolecules. Our analysis found a subtle, quantitatively small but statistically significant, decrease in the diffusion rate of the Val96Ala D2L variant, which bears the amino acid substitution proximal to the ligand-binding site. This observation parallels previously reported reduced affinity of the Val96Ala variant to commonly prescribed neuroleptics, suggesting that there may be a direct link between receptor conformational equilibrium and its intrinsic diffusion dynamics. Interestingly, the absence of the effect of amino acid substitution in the ICL3 on D2L basal lateral mobility underscores the need for further inquiry into the role of the membrane-associated receptor core in defining its dynamic behavior and whether there exists a cause-and-effect relationship between perturbed receptor mobility and receptor-mediated downstream signaling. Additionally, we applied an M&S detection algorithm to determine the relative frequency of D2L-D2L interaction events as defined by diffraction-limited colocalization. Our data showed that a significant fraction (~20%) of D2L receptors transiently colocalized, with no significant differences found for D2L variants examined. Lastly, we implemented a Voronoї tessellation-based algorithm to super-resolved localization maps of mEos2-PH-PLCδ and Qdot-D2L with the goal of uncovering the presence of phospholipid versus receptor nanodomains and globally deciphering the plasma membrane compartmentalization. Our work lays the foundation for future Qdot-based investigation of GPCR nanoscopic organization in human health and disease. We envision that the development of new classes of photoswitchable, compact Qdots with a high photon budget will facilitate investigation of altered neuronal transmembrane protein dynamic behavior in complex, three-dimensional biological specimens. 

## Data Availability

The data presented in this study are openly available in Zenodo at [doi:10.5281/zenodo.5044742], reference number [5044742] (Accessed (deposited) on 30 June 2021).
